# Involvement of Cytochrome P450 1A1 and Glutathione S-Transferase P1 Polymorphisms and Promoter Hypermethylation in the Progression of Anti-Tuberculosis Drug-Induced Liver Injury: A Case–Control Study

**DOI:** 10.1371/journal.pone.0119481

**Published:** 2015-03-23

**Authors:** Lei He, Li Gao, Zhe Shi, Yuhong Li, Lingyan Zhu, Shiming Li, Peng Zhang, Guoying Zheng, Qi Ren, Yun Li, Bo Hu, Fumin Feng

**Affiliations:** 1 Key Laboratory of Occupational Health and Safety, School of Public Health, Hebei United University, Tangshan 063000, China; 2 Department of Clinical Laboratories, Tangshan Tuberculosis Hospital, Tangshan 063000, China; Queen's University Belfast, UNITED KINGDOM

## Abstract

**Background:**

Anti-tuberculosis (anti-TB) drug-induced liver injury (ADLI) is one of the most common adverse effects associated with TB treatment. Cytochrome P450 (CYP) 1A1 and glutathione S-transferase (GST) P1 are important phase I/II metabolizing enzymes involved in drug metabolism and detoxification. Genetic polymorphism and CpG island methylation have been reported as factors influencing the expression of *CYP1A1* and *GSTP1*.

**Objective:**

This study aimed to determine the potential relationships of *CYP1A1* and *GSTP1* polymorphisms and CpG island methylation with ADLI risk.

**Design:**

This was a population-based one-to-one matched case–control study.

**Setting:**

The subjects were patients with TB receiving treatment in China from December 2010 to June 2013.

**Patients:**

In total, 127 patients with TB and ADLI (case group) and 127 patients with TB but without liver injury (control group) were included in this study. Subjects were matched in terms of sex, age, and therapeutic regimen.

**Methods:**

The general condition of each patient was assessed using questionnaires. The *CYP1A1* MspI and *GSTP1* Ile105Val polymorphisms as well as methylation status were detected by polymerase chain reaction (PCR)–restriction fragment length polymorphism and the methylation-specific PCR method.

**Results:**

We found no significant difference in *GSTP1* and *CYP1A1* genotypes between the two groups, probably because the sample size was not large enough; however, patients with ADLI had significantly higher *GSTP1* and *CYP1A1* promoter methylation rates than control subjects [odds ratio (OR) = 2.467 and 2.000, respectively]. After adjusting for drinking, which significantly differed between the groups as per univariate analysis, we found that hypermethylation of *GSTP1* and *CYP1A1* promoters was associated with ADLI (OR = 2.645 and 2.090, respectively).

**Conclusion:**

Hypermethylation of CpG islands of *GSTP1* and *CYP1A1* promoters may thus play important roles in the development of ADLI and provide evidence of being used as novel markers for ADLI risk prediction.

## Introduction

Tuberculosis (TB) is one of the major global health problems, with approximately 8.6 million individuals affected by the disease and 1.3 million deaths worldwide in 2012 alone [1]. Anti-TB drug-induced liver injury (ADLI) is one of the most significant and serious adverse effects of TB treatment. The incidence of ADLI varies from 5.0% to 33.0% in different populations [2, 3]. Such an adverse effect of the TB treatment significantly hampers global TB epidemic control. Therefore, the mechanism by which ADLI occurs must be elucidated to improve the curative rate of TB.

Anti-TB drugs are metabolized mainly by drug-metabolizing enzymes (DME) such as phase I enzymes [e.g., cytochrome P450 (CYP)], which help bioactivate toxic substances, and phase II enzymes [e.g., glutathione S-transferase (GST)], which can reduce the toxicity of electrophilic compounds formed by phase I enzymes [4]. Hence, the actual effects of the accumulation of toxic metabolic products of drugs and the detoxification process depend on the balance between phase I and phase II enzymes [5].

ADLI is caused by a heterogeneous mix of factors; evidence suggests that the interplay between genetic and epigenetic events has significant implications in the pathogenesis of ADLI. Drug enzymes can undergo genetic or epigenetic alterations, resulting in changes in or complete loss of their activity, thereby leading to the impairment of cellular detoxification and, consequently, to liver diseases [6]. Evidence suggests that decreased expression of GSTP1 or CYP1A1 induced by polymorphisms at their respective gene loci has been associated with many liver diseases such as hepatocellular carcinoma [7–10]. In addition, decreased GSTP1 and CYP1A1 expression induced by methylation of the *GSTP1* and *CYP1A1* promoters, respectively, has been reported in many liver diseases such as acute-on-chronic hepatitis B liver failure [11–14]. These findings suggest that polymorphisms or hypermethylation of *GSTP1* and *CYP1A1* may be important in ADLI.

In the present study, we investigated the effects of *GSTP1* and *CYP1A1* polymorphisms and changes in promoter CpG island methylation on the development of ADLI in patients with TB.

## Materials and Methods

### Ethics statement

All the patients provided their verbal informed consent to participate in this study, and we obtained an approval letter from the ethics committee of Hebei United University; the approval number is 10–007. Informed consent for the blood remaining to be used for further research was verbally obtained during the epidemiological survey. We explained the content and purpose of the study to the patients, and if the patients agreed, we selected “yes” to record their consent. This consent procedure was approved by the ethics committee because we only used the remaining blood and there was no damage to the patients’ interests.

### Patients and controls

We used a one-to-one matched case–control design, which recruited patients diagnosed with TB from December 2010 to June 2013 in Tangshan Tuberculosis Hospital (China). The study participants included 127 patients with TB and ADLI (case group) and 127 patients with TB but without liver injury (control group). All the patients were initially subjected to TB treatment. The hospital is designated as a TB treatment unit by the government of Tangshan City; therefore, we could reduce selection bias to some extent.

The criteria for inclusion in the case group included the occurrence of liver injury after 6 months of anti-TB drug therapy. ADLI was defined according to the Danan criteria promulgated in 1990 [15, 16]. The inclusion criteria for the control group included the absence of liver injury after 6 months of anti-TB drug therapy and a match with patients in the case group in terms of age (<5 years difference), sex, and therapeutic regimen (daily 2S(E)HRZ4HR: S, streptomycin; E, ethambutol; H, isoniazid, R, rifampicin; Z, pyrazinamide; dose increased for 2 months and then consolidated for 4 months).

This study included 74.02% men (94 pairs) and 25.98% women (33 pairs). In the case group, the maximum age was 86 years, the youngest patient was 17 years old, and the mean age was 48.98 years, whereas in the control group, the maximum age was 88 years, the youngest subject 20 years old, and the mean age 49.13 years. Age and sex were matched to ensure comparability of the case and control groups.

The exclusion criteria were as follows: presence of abnormal liver function before the administration of TB treatment; co-occurrence of other diseases that can cause liver function abnormalities such as viral hepatitis, alcoholic liver disease, autoimmune hepatitis, and hypoxemia; and consumption of other drugs that can cause liver dysfunction in patients.

### Sample size

The sample size was estimated as recommended by Schlesselman, as follows [17]:

$$\begin{array}{l} m={\left[z_{\alpha }/2+z_{\beta }\sqrt{p\left(1-p\right)}\right]}^{2}/{\left(p-1/2\right)}^{2}\\ p=OR/\left(1+OR\right)\approx RR/\left(1+RR\right) \end{array}$$ (1)

$$\begin{array}{l} M\approx m/\left(p_{0}q_{1}+p_{1}q_{0}\right)\\ p_{1}=p_{0}RR/\left[1+p_{0}\left(RR-1\right)\right]\\ q_{1}=1-p_{1}\\ q_{0}=1-p_{0}\;, \end{array}$$ (2)

where m represents the number of inconsistent results; p_1_ represents an estimation of the exposure rate in the case group; and p_0_ represents an estimation of the exposure rate in the control group. We assumed p_0_ to be 30% and the odds ratio (OR) to be 2.5 in the present study. The sample size was calculated with 90% power (1-β) and 5% significance (α), Z_α_ = 1.96, Z_*β*_ = 1.28.

### Epidemiological investigation

The patients’ general condition and other basic information were obtained through an epidemiological survey during the study period, which included sex, age, height, weight, marital status, education, profession, smoking, drinking, past medical history, therapeutic regimen, and liver function examination results. The relevant items are defined as follows: 1. Smoking: the advice on the smoking survey method standard recommended by WHO (1984) was used, in which a subject who smoked more than one cigarette per day for more than a year was defined as a smoker. 2. Drinking: a subject who drank at least 50 g of alcohol at least twice a week and continued for more than a year was defined as a drinker.

### Plasma/blood cell collection and genomic DNA extraction

Plasma/blood cells were obtained from the peripheral blood by centrifugation and stored at −80°C for detection. The salting-out method was used to extract genomic DNA. A serum-free DNA extraction kit (magnetic particles) was used to extract plasma-free DNA.

### Genotyping

Polymerase chain reaction–restriction fragment length polymorphism (PCR–RFLP) analysis was performed to detect the genotypes of *CYP1A1* (MspI) and *GSTP1* (Ile105Val). The primer sequences used in the present study are listed in Table 1. DNA from blood samples from the case and control subjects was isolated and amplified using *CYP1A1* and *GSTP1* primers. PCR products were then digested with MspI/BsmA I. Following this, the digested PCR products were run on 2% agarose gel, and at least 10% of the samples were subjected to the same procedure to confirm the results. Genotyping method: PCR products for the *GSTP1* heterozygous genotypes (A/G) were 452-, 222-, and 230-bp long, while those for the *GSTP1* homozygous mutant genotypes (G/G) were 222- and 230-bp long. The *GSTP1* wild-type homozygous genotype (A/A) was 452-bp long. PCR products for the *CYP1A1* heterozygous genotypes (C/A) were 340-, 200-, and 140-bp long. The *CYP1A1* homozygous mutant genotypes (A/A) were 200- and 140-bp long, and the *CYP1A1* wild-type homozygous genotype (C/C) was 340-bp long.

**Table 1:** Polymorphism and Methylation-Specific Primer Sequences for ***CYP1A1*** and ***GSTP1*.**

Oligonucleotide name	Primer sequence
CYP1A1 Msp I (Forward primer)	5'-CAGTGAAGAGGTGTAGCCGCT-3'
CYP1A1 Msp I (Reverse primer)	5'-TAGGAGTCTTGTCTCATGCCT-3'
GSTP1 Ile105Val (Forward primer)	5'-CATCCTTCCACGCACATCCTC-3'
GSTP1 Ile105Val (Reverse primer)	5'-CGTTACTTGGCTGGTTGATGTCC-3'
CYP1A1 Unmethylated (Forward primer)	5'-GGATTATTTTTTGGTTTGGATTAGT-3'
CYP1A1 Unmethylated (Reverse primer)	5'-AACCTAACTACCTACCTCCAACACT-3'
CYP1A1 Methylated (Forward primer)	5'-GATTATTTTTTGGTTTGGATTAGC-3'
CYP1A1 Methylated (Reverse primer)	5'-TAACCTAACTACCTACCTCCGACG-3'
GSTP1 Unmethylated (Forward primer)	5'-AAGGTTAGGAGTTTGAGATTAGTTTG-3'
GSTP1 Unmethylated (Reverse primer)	5'-CCTCCCAAATAAATAAAATTATAAATACA-3'
GSTP1 Methylated (Forward primer)	5'-AGGTTAGGAGTTCGAGATTAGTTC-3'
GSTP1 Methylated (Reverse primer)	5'-CCCGAATAAATAAAATTATAAATACGTA-3'

### Bisulfite treatment of genomic DNA and methylation-specific PCR

Genomic DNA isolated from plasma was modified with sodium bisulfite using an EZ DNA methylation-gold kit (ZYMO Research Corporation, Irvine, CA, USA). The methylation-specific PCR (MSP) method was used to detect the methylation levels of *GSTP1* and *CYP1A1* in plasma-free DNA. The primer sequences used are listed in Table 1. The MSP mixture sample was incubated for 5 min at 95°C, followed by 60 cycles of denaturing at 95°C for 30 s, annealing at 66°C to 56°C for 30 s, extension at 72°C for 30 s (annealing temperature was decreased by 1°C at an interval of two cycles up to 56°C), and final extension at 72°C for 7 min. A negative sample (no DNA) was used in each PCR set. PCR products were stained, observed under UV illumination, and analyzed on 3% agarose gels. The products of methylated and unmethylated primers of *CYP1A1* were both 194 bp in size; the products of the methylated and unmethylated primers of *GSTP1* were 108 and 113 bp in size, respectively.

Samples pertaining to matched cases and controls were analyzed in the same batch, and laboratory personnel were unable to distinguish between cases and controls.

### Statistical analysis

Univariate and multivariate analyses of risk factors used conditional logistic regression to compare patients with ADLI with their matched controls for general factors, including marital status, education, profession, body mass index (BMI), smoking, drinking, *CYP1A1* and *GSTP1* genotypes, and methylation status. ORs and 95% confidence intervals (CIs) were calculated to determine the relationship between the risk factor and ADLI. Statistical analyses were performed using SPSS for windows, version 17.0. *P* < 0.05 was considered to be statistically significant.

## Results

### Basic characteristics of the subjects

We collected a total of 2683 patients with TB from December 2010 to June 2013; the number of patients who accepted the whole standardized hospitalization for 6 months was 1897; 1458 (76.86%) of these received initial treatment, whereas 439 (23.14%) were re-treated. All the patients were initially subjected to TB treatment. Of the 1458 cases, 175 patients (12%) were diagnosed with ADLI on the basis of the inclusion and exclusion criteria. We calculated the sample size on the basis of the equations recommended by Schlesselman, eventually selecting 127 pairs of patients for the study.

Owing to the one-to-one individual matching procedure, there was no statistical difference in the distribution of age or sex between cases and controls. Table 2 summarizes other basic characteristics of subjects in the case and control groups. We found that the distributions of drinking, *CYP1A1* methylation, and *GSTP1* methylation were significantly different between the case and control groups.

**Table 2:** Conditional logistic regression analysis of ADLI-influencing factors.

Factors		*Case group (n)*	*Control group (n)*	*P-value*	*OR*	*95% CI*
Univariate analysis*						
Marital status	Married	108 (85.04%)	100 (78.74%)	0.082	2.333	0.897–6.072
	Unmarried	19 (14.96%)	27 (21.26%)			
Education	Senior	34 (26.77%)	36 (28.35%)	—	ref.	—
	Junior	57 (44.88%)	57 (44.88%)	0.604	0.835	0.423–1.649
	Primary	36(28.35%)	34(26.77%)	0.780	0.907	0.456–1.802
Profession	Others	47 (37.01%)	44 (34.65%)	—	ref.	—
	Worker	27 (21.26%)	26 (20.47%0	0.865	1.059	0.545–2.060
	Farmer	53 (41.73%)	57 (44.88%)	0.931	1.026	0.580–1.813
BMI	BMI < 18.5 kg/m^2^	23 (18.11%)	18 (14.17%)	0.386	1.357	0.680–2.707
	BMI ≥ 18.5 kg/m^2^	104 (81.89%)	109 (85.83%)			
Smoking	Yes	35 (27.56%)	37 (29.13%)	0.768	0.917	0.514–1.635
	No	92 (72.44%)	90 (70.87%)			
Drinking	Yes	40 (31.50%)	29 (22.83%)	0.037	2.083	1.047–4.147
	No	87 (68.50%)	98 (77.17%)			
CYP1A1 polymorphism	Wild genotype	65 (51.18%)	74 (58.27%)	0.293	1.281	0.807–2.034
	Mutant genotype**	62 (48.82%)	53 (41.73%)			
GSTP1 polymorphism	Wild genotype	70 (58.12%)	88 (69.29%)	0.065	1.552	0.973–2.475
	Mutant genotype	57 (48.88%)	39 (30.71%)			
CYP1A1 methylation	Methylated***	105 (82.68%)	89 (70.08%)	0.024	2.000	1.097–3.645
	Unmethylated	22 (11.76%)	38 (29.92%)			
GSTP1 methylation	Methylated	103 (81.10%)	81 (63.78%)	0.003	2.467	1.354–4.494
	Unmethylated	24 (18.90%)	46 (36.22%)			
*Multivariate analysis*						
Drinking				0.038	2.176	1.046–4.526
GSTP1 methylation				0.002	2.645	1.414–4.946
CYP1A1 methylation				0.023	2.090	1.107–3.946

*Conditional logistic regression method was used for univariate analysis, which was suitable for the matching study.

**Mutant genotype combined the heterozygous mutant genotypes and homozygous mutant genotypes together.

***Methylated combined the complete methylated and partial methylated groups together.

### Univariate analysis

We analyzed the distribution of risk factors in the two groups using the conditional logistic regression method, including marital status, education, profession, BMI, smoking, drinking, *GSTP1* and *CYP1A1* polymorphisms, and methylation of *GSTP1* and *CYP1A1*. We found that drinking and methylation levels of CpG islands of *GSTP1* and *CYP1A1* significantly differed between the two groups. The frequencies of drinking were 31.50% and 22.83% in the case and control groups, respectively. The frequencies of methylated CpG islands of the *GSTP1* promoter were 81.10% and 63.78% in the case and control groups, respectively, while those of *CYP1A1* were 82.68% and 70.08%, respectively. Drinking and hypermethylation of the *GSTP1* and *CYP1A1* promoters were associated with ADLI (drinking: OR = 2.083, 95% CI = 1.047–4.147; *GSTP1* methylation: OR = 2.467, 95% CI = 1.354–4.494; *CYP1A1* methylation: OR = 2.000, 95% CI = 1.097–3.645). Detailed results are shown in Table 2.

### Multivariate analysis

To eliminate confounding effects, we included all potential confounding factors, including those that had *P <* 0.05 in univariate analysis, in a multivariate analysis model. After adjusting for drinking, we found that hypermethylation of *GSTP1* and *CYP1A1* was a risk factor for the development of ADLI, with adjusted ORs of 2.645 and 2.090, respectively (Table 2).

## Discussion

In the present study, no associations were observed between *GSTP1* and *CYP1A1* polymorphisms and ADLI risk; however, our findings indicate that hypermethylation of *GSTP1* and *CYP1A1* is related to ADLI; the hypermethylated genes may play important roles in the development of ADLI.

We report the methylation status and polymorphisms of *GSTP1* and *CYP1A1* in 127 pairs of patients with TB with matched sex, age, and therapeutic regimen. Unlike previous studies that had a group case–control design, the present study was designed with therapeutic regimen as the matching factor to reduce individual differences in ADLI progression caused by different therapeutic regimens.

Previous studies have shown that many factors, including BMI, alcohol, sex, and age as well as chronic hepatitis B, hepatitis C virus, or HIV infection, are associated with the occurrence of ADLI [18]. To determine whether the presence of risk factors may increase the risk of ADLI, we compared the distribution of several factors, including marital status, education, profession, BMI, smoking, and drinking, between the case and control groups. Our findings suggest that drinking is a risk factor for ADLI in patients with TB. One explanation for this is that ethanol induces various metabolic enzymes in vivo [19]: high ethanol concentrations in long-term drinkers may cause increased metabolic activity and elevated production of toxic metabolic waste, thereby increasing the risk of ADLI.

Previous studies have reported that mutations in several drug-metabolism genes, such as polymorphisms in *CYP2E1*, *GSTM1*, *GSTT1*, *NAT2*, and *UGT*s, constitute risk factors for ADLI; however, the results have not been entirely consistent. In general, individuals with the *CYP2E1* C1/C1 genotype, slow acetylator *NAT2* genotype, or *GSTM1* null genotype have been shown to have an increased risk of ADLI [20, 21]. In addition, studies have shown the Ile105Val and MspI polymorphisms in *GSTP1* and *CYP1A1*, respectively, with mutant genotypes likely reducing the activities of the respective genes [22, 23]. However, we found *GSTP1* and *CYP1A1* polymorphisms to be unrelated to increased risk of ADLI. This result may be because of an insufficient sample size and should be addressed by increasing the number of cases in future studies.

A previous research suggested that activation or silencing of certain signaling pathways plays a major role in ADLI development. Genetic information is carried not only in DNA sequences but also in epigenetic variations [24]. “Epimutations”, including DNA methylation, may occur more frequently than gene mutations and may affect ADLI. Previous studies examined rat hepatocyte DNA on a genome-wide scale in addition to *CYP2E1* promoter methylation and showed that both genome-wide methylation and *CYP2E1* methylation status were related to isoniazid-induced liver injury in clinical experiments involving different populations [25, 26]. Kovalenko [27] demonstrated abnormal changes in CpG island methylation of the *GSTP1* promoter in pyrazinamide-induced rat liver injury. Other studies have also shown that the suppression of GSTP1 expression caused by promoter methylation contributes to the early stage of hepatocellular carcinoma [11, 28, 29]. To determine whether methylation is implicated in genetic susceptibility to ADLI, we compared the methylation status of *GSTP1* and *CYP1A1*. We found that compared with the control group, the promoters of both the genes were hypermethylated in patients with ADLI. The frequencies of methylated CpG islands of the *GSTP1* promoter were 81.10% and 63.78% in the case and control groups, respectively, while those of *CYP1A1* were 82.68% and 70.08%, respectively. Thus, both the genes had a high degree of methylation in their promoter regions. These findings indicate that most hypermethylation events in the promoter region occurred in the CpG islands in ADLI. Notably, our findings also revealed that patients with hypermethylated CpG islands of *GSTP1* and *CYP1A1* manifested a higher risk of ADLI development than those with hypomethylated CpG islands (OR = 2.467, 2.000). Gene promoter methylation is generally related to transcriptional repression via mechanisms such as direct prevention of transcription factor binding to DNA binding sites [30] or via complex indirect mechanisms such as chromatin remodeling [31–33]. For instance, DNA methylation can recruit methyl-CpG-binding domain proteins, which in turn can recruit histone-modifying and chromatin-remodeling complexes to methylated sites, thereby inhibiting gene expression. In addition, gene promoter methylation is catalyzed by methyl transferase enzymes, including DNMT1, DNMT2, DNMT3a, and DNMT3b [34]. It has previously been reported that DNMT1, DNMT3a, and DNMT3b are expressed in various tissues of the human body, including the liver [35]. Therefore, toxic metabolites of anti-TB drugs may induce the methylation of the promoter regions of *GSTP1* or *CYP1A1* by increasing the activity of DNMT1, DNMT3a, and DNMT3b. This hypothesis needs to be further validated with future studies.

To eliminate the effects of confounding factors, we included all potential confounding factors in a multivariate logistic regression model. The results showed that after adjusting for drinking, hypermethylation of the CpG islands of *GSTP1* and *CYP1A1* promoters were bona fide risk factors for ADLI. Thus, the data presented here suggest that methylation causes the disruption of *GSTP1* and *CYP1A1* function, eventually leading to liver injury.

To the best of our knowledge, the occurrence and development of ADLI involves multiple factors associated with changes in multiple genes and multiple processes. Considering that genetic mutations and epigenetic changes are two of the most important factors that determine ADLI development and that both epigenetic and genetic alterations may affect the expression of GSTP1 and CYP1A1, studying the effects of the combination of polymorphism and methylation may provide a more robust evaluation than examining individual factors. In the present study, combined associations were observed between the methylation status and polymorphism of *GSTP1* and *CYP1A1*, which indicate that the combination of polymorphism and methylation is a highly important risk factor for ADLI, with an OR value four times higher than the maximum effect of a single gene.

In the present study, we found no significant difference in *GSTP1* and *CYP1A1* genotypes between the two groups, probably because the sample size was not large enough. Despite the fact that the relationships between *GSTP1* and *CYP1A1* promoter hypermethylation and ADLI were observed well, 127 pairs is not sufficient to completely explore the role of gene polymorphisms; however, this study provides some evidence that hypermethylation of *GSTP1* and *CYP1A1* promoters may be potential biomarkers for the early diagnosis and prevention of ADLI.

Unlike genetic alterations such as mutations and deletions, epigenetic changes have the potential to be reversed. Several clinical trials are underway to evaluate the potential for cancer prevention and therapy by reversing methylation-induced alterations [36–38]. Based on our findings, it is highly likely that patients with TB who harbor hypermethylation changes in *GSTP1* and *CYP1A1* promoter CpG islands are at a high risk of liver damage. DNA demethylation drugs have the potential to reverse this change, allowing re-expression of drug-metabolism enzymes. When they are used in a timely manner in combination with traditional drugs, it is possible to reduce or even avoid the occurrence of liver injury during TB treatment. Although many problems related to the clinical application of demethylation drugs remain to be overcome, further improvements in such drugs may bring optimistic outlooks for the diagnosis and treatment of diseases.

## Conclusion

In conclusion, no associations between *GSTP1* and *CYP1A1* polymorphisms and ADLI risk were observed in the present study. However, our findings indicate that hypermethylation of *GSTP1* and *CYP1A1* may be important in the development of ADLI and may prove useful as novel markers for ADLI risk prediction.
